# Detecting pelvic fracture on 3D-CT using deep convolutional neural networks with multi-orientated slab images

**DOI:** 10.1038/s41598-021-91144-z

**Published:** 2021-06-03

**Authors:** Kazutoshi Ukai, Rashedur Rahman, Naomi Yagi, Keigo Hayashi, Akihiro Maruo, Hirotsugu Muratsu, Syoji Kobashi

**Affiliations:** 1 Research and Development Center, GLORY Ltd, Himeji, Japan; 2 Graduate School of Engineering, University of Hyogo, Himeji, Japan; 3 Himeji Dokkyo University, Himeji, Japan; 4 Steel Memorial Hirohata Hospital, Himeji, Japan

**Keywords:** Machine learning, Orthopaedics, Trauma

## Abstract

Pelvic fracture is one of the leading causes of death in the elderly, carrying a high risk of death within 1 year of fracture. This study proposes an automated method to detect pelvic fractures on 3-dimensional computed tomography (3D-CT). Deep convolutional neural networks (DCNNs) have been used for lesion detection on 2D and 3D medical images. However, training a DCNN directly using 3D images is complicated, computationally costly, and requires large amounts of training data. We propose a method that evaluates multiple, 2D, real-time object detection systems (YOLOv3 models) in parallel, in which each YOLOv3 model is trained using differently orientated 2D slab images reconstructed from 3D-CT. We assume that an appropriate reconstruction orientation would exist to optimally characterize image features of bone fractures on 3D-CT. Multiple YOLOv3 models in parallel detect 2D fracture candidates in different orientations simultaneously. The 3D fracture region is then obtained by integrating the 2D fracture candidates. The proposed method was validated in 93 subjects with bone fractures. Area under the curve (AUC) was 0.824, with 0.805 recall and 0.907 precision. The AUC with a single orientation was 0.652. This method was then applied to 112 subjects without bone fractures to evaluate over-detection. The proposed method successfully detected no bone fractures in all except 4 non-fracture subjects (96.4%).

## Introduction

Pelvic fracture can be considered as a significant health concern, representing one of the most common causes of hospitalization and mobility loss^1^. Moreover, pelvic fracture is a key cause of mortality in the elderly^1–4^. The number of patients with pelvic fracture is continuously increasing among elderly populations in various countries, including Japan and the United States^5–9^. Quick and precise diagnosis is required in the hospital, especially in emergency departments, to enable early surgical intervention and preservation of the functionality of joints and quality of life^10,11^. This increase in patients is leading to an increasing load on radiologists, contributing to initial misdiagnoses^12^. Such misdiagnoses result in worsened prognosis, increased costs of treatment, and elevated mortality rates^2,13^.

Pelvic fractures are more perceptible on images from computed tomography (CT)^14,15^, which are widely used to diagnose pelvic fractures. As CT data usually contain a large number of images, a substantial investment in time is required to interpret each of the images to identify fractures, which then carries a risk of overlooking fractures^16^. An automated system to detect pelvic fractures from CT may thus assist physicians to diagnose fractures. Further, such results can be applied to augmented reality (AR) to assist surgeons in complex surgical procedures^17^.

Several methods have been proposed to automatically detect pelvic fractures on CT. Chowdhury et al.^18^ introduced some methods of pelvic fracture detection based on graph cut theory, curvatures, morphological analysis, and their combinations. That study detected fractures by evaluating discontinuities or gaps in the pelvic bone. However, natural gaps exist between pelvic bones and could be incorrectly detected as fractures and thus increase the number of false-positive results. Another method was proposed to detect fractures on CT images of traumatic pelvic injuries based on the registered active shape model and 2D stationary wavelet transform^19^. Accuracy, sensitivity, and specificity in 12 subjects were 91.98%, 93.33%, and 89.26%, respectively. That method focused only on completely displaced bone fractures, and did not discuss incompletely displaced fractures or compression fractures. The number of subjects was also limited.

Some studies have detected various kinds of bone fractures on 2D X-ray radiographs based on deep convolutional neural networks (DCNNs). Lindsey et al.^20^ proposed a method of wrist fracture detection. This method estimated a conditional probability map which represents a probability of fracture at each pixel. Thian et al.^21^ proposed a method to detect wrist fractures using frontal or lateral X-ray radiographs based on faster region-based convolutional neural network (Faster R-CNN) architecture. Detection accuracies in frontal and lateral radiographs of the wrist were 88.9% and 91.2%, respectively. A method to detect intertrochanteric hip fractures from X-ray radiographs of the femoral head and the greater and lesser trochanters was proposed based on VGG_16^22^, a kind of DCNN. Detection accuracy was reported as 95.5%, higher than the detection accuracy of orthopedic surgeons (92.2%). Sato et al.^23^ introduced a CNN based method to detect hip fracture on plain X-ray radiograph. The experimental results from 300 images showed that the accuracy, sensitivity, specificity, F-value, and area under the curve (AUC) were 96.1%, 95.2%, 96.9%, 0.961, and 0.99, respectively. Cheng et al.^24^ developed a human-algorithm integration system to improve the diagnosis of hip fracture. Another method to classify proximal femur fracture from X-ray images was proposed based on a multistage architecture of successive CNNs in cascade along with gradient class activation maps (Grad-CAM) to visualize the most relevant areas of the images^25^. Mean accuracies of the method for 3-class and 5-class classifications were 0.86 and 0.81 respectively. The proposed CAD system based on the method improved accuracy of specialists by 14%. However, these methods were based on 2D images, and could not be applied directly to 3D images.

As related studies involving fractures at sites other than the pelvis, a few studies have proposed methods of automated bone fracture detection using CT. Bar et al.^26^ proposed a method to detect vertebral compression fractures (VCFs), based on DCNN and long short-term memory (LSTM). This method first estimated a vector of probabilities from patches of CT images using DCNN, then classified these patches into VCF using LSTM. Accuracy, sensitivity, and specificity were 89.1%, 83.9%, and 93.8%, respectively. Roth et al.^27^ proposed a method to detect posterior element fractures from CT images based on ConvNet. Sensitivities at 5 false positives per patient (FP/P) and 10 FP/P were 71% and 81%, respectively. Recently, Zhou et al. proposed an automatic method to detect and classify rib fractures on thoracic CT^28^. This method is based on Faster R-CNN. With the assistance of this method, the sensitivity for diagnosing rib fractures was increased by 23.9%.

Those papers mainly focused on detecting bone fractures in 2D spaces. The methods used CT, but did not segment 3D fracture regions, and did not consider 3D image features and structure. Basically, those methods cannot evaluate the 3D spatial connectivity of fractures. A straightforward approach to evaluating 3D information is 3D-DCNN^29^, but as the availability of 3D data is limited, a suitable 3D-DCNN model to detect fractures is not yet known, and the method would be computationally costly. Another approach is to synthesize 2D images from 3D volume data, known as 2.5D representation. Such 2.5D representation has been applied to lymph node detection using CT images^30^ and cerebral aneurysm detection on Magnetic Resonance (MR) angiography^31^. The 2.5D approach synthesizes 2D images from 3D volume data in orthogonal and diagonal directions. The synthesized 2D images may contain a large amount of 3D information in comparison with the original raw 2D images.

Bone fractures on CT images can take various appearances. Any surface displaced due to bone fracture and vertical to the imaging plane will be clearly apparent. However, fracture surfaces displaced parallel to the imaging plane can be hard to recognize. This means that appropriate orientation of the imaging plane is crucial. However, the appropriate orientation cannot be determined initially, because each fracture has a different orientation, and acquisition of images in multiple orientations from the same patient is unfeasible because of the risks associated with X-ray exposure. The present study addressed this obstacle by reconstructing raw sectional images into multiple-orientated images. We assumed that detection accuracy in 3D space would thus be improved by detecting fractures in the reconstructed multiple-orientated images simultaneously and aggregating those in 3D space.

This study proposed a fully automated method of fracture detection on 3-dimensional computed tomography (3D-CT) of pelvic region. The proposed method is based on multiple 2D-DCNNs, in which each 2D-DCNN evaluates images in a different orientation. This utilizes YOLOv3^32^, a real-time object detection system, to detect fractures on 2D images. For each orientation, three 2.5D slab images are synthesized with three different thicknesses. Fracture candidates are detected by each YOLOv3 model with different orientations simultaneously. The 3D fracture region is finally detected by integrating fracture candidates. By detecting bone fractures in multiple orientations, the proposed method improves detection accuracy. The proposed method was validated using two datasets: one dataset of 93 subjects with bone fractures (dataset **A**), and another dataset of 112 subjects with no bone fractures (dataset **B**). Results were evaluated by comparison with the ground truth fractures, AUC, recall and precision were evaluated fracture-wise using dataset **A**. The AUC was 0.824 for an intersection of union (IoU) of 10%. Recall and precision at the highest F score were 0.805 and 0.907 respectively. Specificity was calculated subject-wise using dataset **B** and the specificity was 0.964 (4 of 112 subjects).

## Results

### Conceptual representation of the proposed method

**Figure 1:**
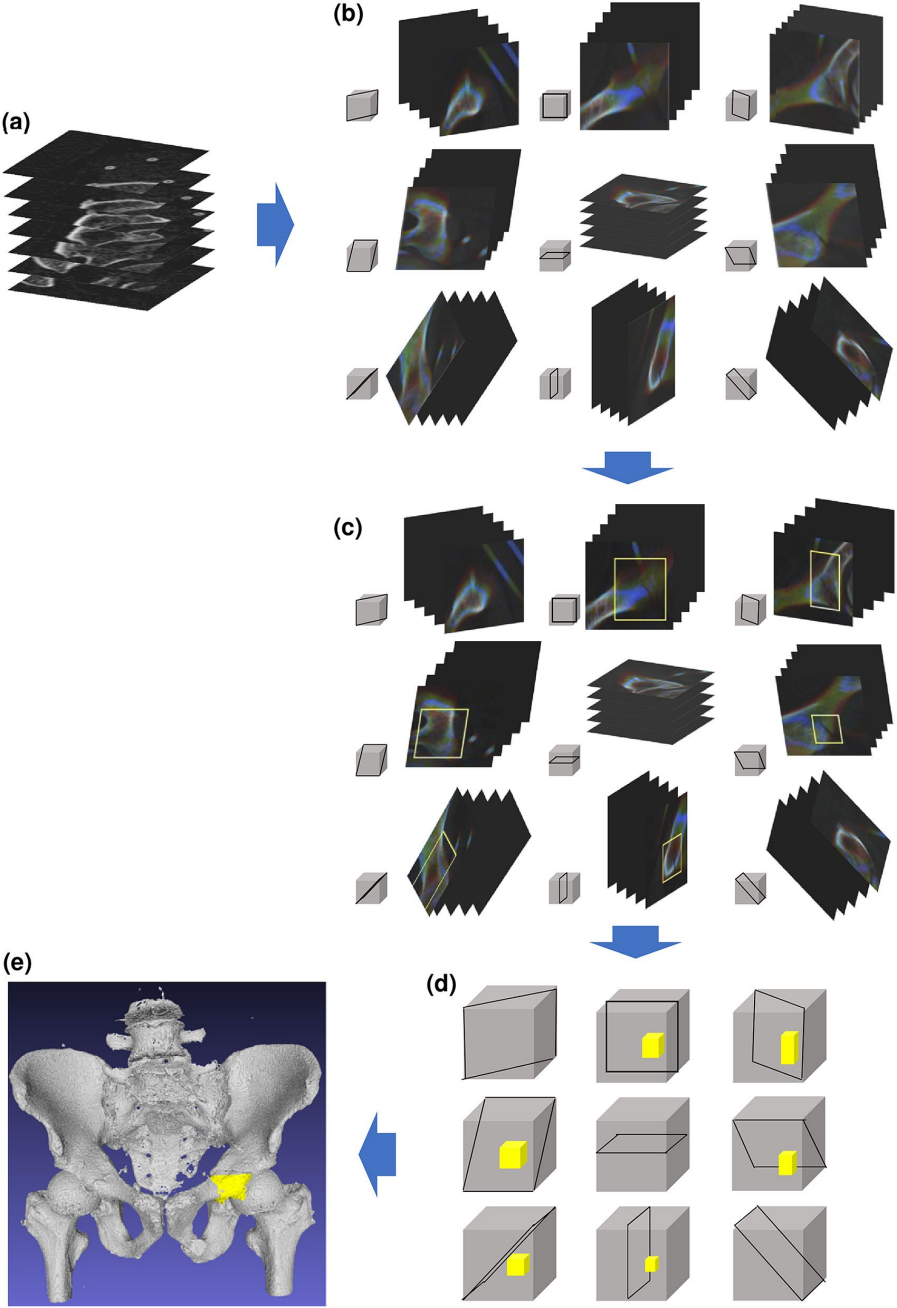
Conceptual diagram of the proposed method. (**a**) A series of axial CT images obtained from a subject. Each image represents 50 × 50-mm area for easy understanding. (**b**) Nine synthesized, orientated 2.5D images. Three slab images with thicknesses of 18.6 mm, 9.0 mm, and 0.6 mm are visualized by R-G-B colors, respectively. (**c**) Detection of 2D fracture candidates. (**d**) Thickening of 2D fracture candidates. (**e**) Fracture region detection.

A conceptual diagram of the proposed method is illustrated in Fig. 1. The method first synthesizes 2.5D slab images with thicknesses of 18.6 mm, 9.0 mm, and 0.6 mm in nine orientations (Fig. 1b) from the provided CT images (Fig. 1a). Second, the method detects fracture candidates for each orientation using YOLOv3 model simultaneously (Fig. 1c). Third, 3D volumes of fracture candidates are formed by thickening the detected 2D boundary box (Fig. 1d). Finally, the 3D fracture region is determined by integrating fracture candidates (Fig. 1e).

The method has two parameters, ${C}_{th}$ and ${I}_{th}$. ${C}_{th}$ represents a threshold of confidence score to detect the 2D bounding box with YOLOv3 model. The confidence score takes a value between 0 and 1, with higher values showing higher confidence. Bounding boxes with confidence scores equal to or exceeding ${C}_{th}$ are detected. ${I}_{th}$ represents the threshold for the degree of fracture. When the number of orientations in which the voxel is included in fracture candidates equals or exceeds this threshold, the voxel is extracted as a fracture voxel.

### Detection of 3D fracture regions

**Figure 2:**
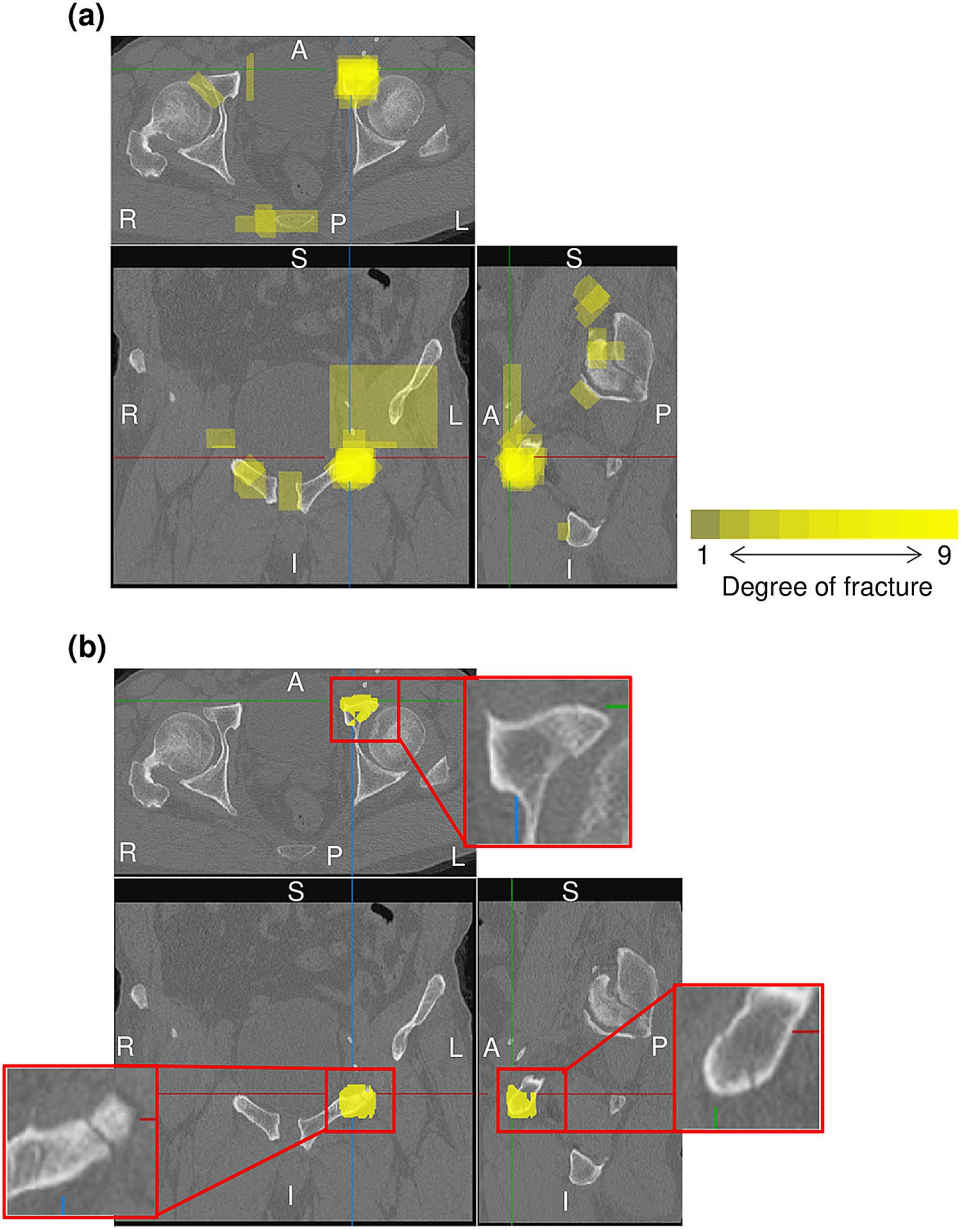
Estimated degree of fracture on multiplanar reconstruction images. Top: axial image; bottom-left: coronal image; bottom-right: sagittal image. L: left; R: right; A: anterior; P: posterior; S: superior; I: inferior. (**a**) Integrated 3D fracture candidate region overlapping on CT images (${C}_{th}$: 0.2). Yellow represents the degree of fracture. (**b**) Resultant 3D fracture region (${I}_{th}$: 6). Yellow represents the detected region. The enlarged image shows raw CT images for the detected fracture region.

Figure 2a shows the estimated degree of fracture overlaid on multiplanar reconstruction images with ${C}_{th}$ of 0.2. The degree of fracture is estimated at each voxel, and assumes a value between 0 and 9 as the number of orientations under evaluation; 0 means that no fracture is detected in any orientation, and 9 means that fracture is detected in all orientations. Figure 2b shows the resultant fracture region with ${I}_{th}$ = 6. Over-detection occurring in the individual orientation detection step is suppressed by aggregation of fracture candidates for each orientation.

### Detection accuracy

**Figure 3:**
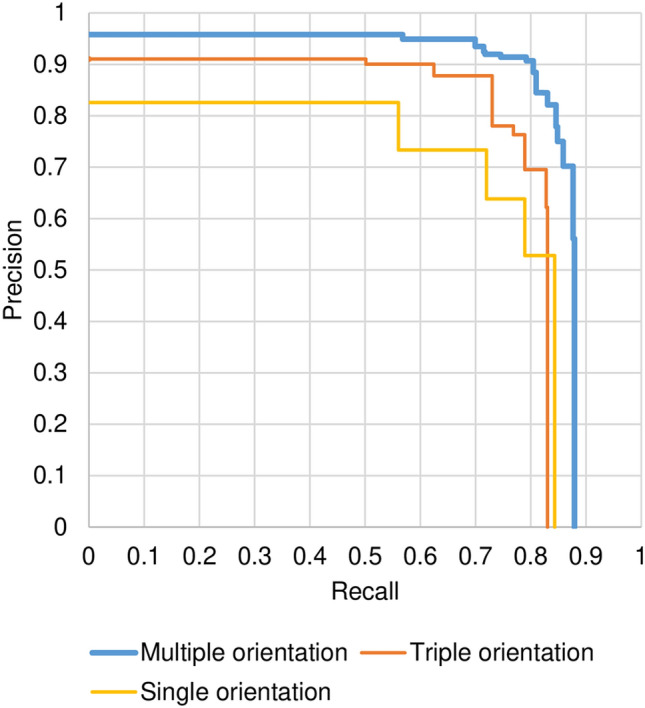
Precision-recall curve.

Dataset **A** with bone fractures was used to evaluate the proposed method, and sixfold cross-validation was conducted. Dataset **A** included 93 subjects with 389 fractures. All subjects were divided into 6 groups, with 5 groups used for training, and the remaining group used for evaluation. To evaluate performance, the IoU between the detected fracture region and ground truth fracture region was calculated. Detection accuracy was evaluated for each fracture, and the evaluation metrics were precision, recall, and AUC. The threshold used for IoU was 10%. Figure 3 shows the interpolated precision-recall (PR) curve, obtained using the set of parameters as combinations of ${C}_{th}$ = 0.01, 0.02, 0.05, 0.1, 0.2, 0.4, 0.6, 0.8 and ${I}_{th}$ = 3, 4, 5, 6, 7, 8, 9. The AUC for multiple orientations was 0.824 with an IoU of 10%.

To demonstrate the effectiveness of the proposed method using multiple orientations, single-orientation detection, and triple-orientation results are also plotted in Fig. 3. The single-orientation method detected fractures using only axial images, and the triple-orientation method used axial, coronal, and sagittal images. AUCs with the single- and triple-orientation methods were 0.652 and 0.734, respectively. The proposed method detected bone fractures successfully using more orientations, and the less-orientated method failed when the fracture did not appear clearly in the given orientation. We concluded that multiple-orientated analysis is quite effective to detect bone fractures from CT images.

Parameters ${C}_{th}$ and ${I}_{th}$ should be optimized to provide the highest value of the two evaluation metrics “recall” and “precision”, although a tradeoff exists between recall and precision. F score was therefore used to evaluate the overall performance of the proposed method. The highest F score for an IoU of 10% was 0.853 when ${C}_{th}$ was 0.2 and ${I}_{th}$ was 6. Recall was 0.805 and precision was 0.907.

### 3D visualization of the detected fractures

**Figure 4:**
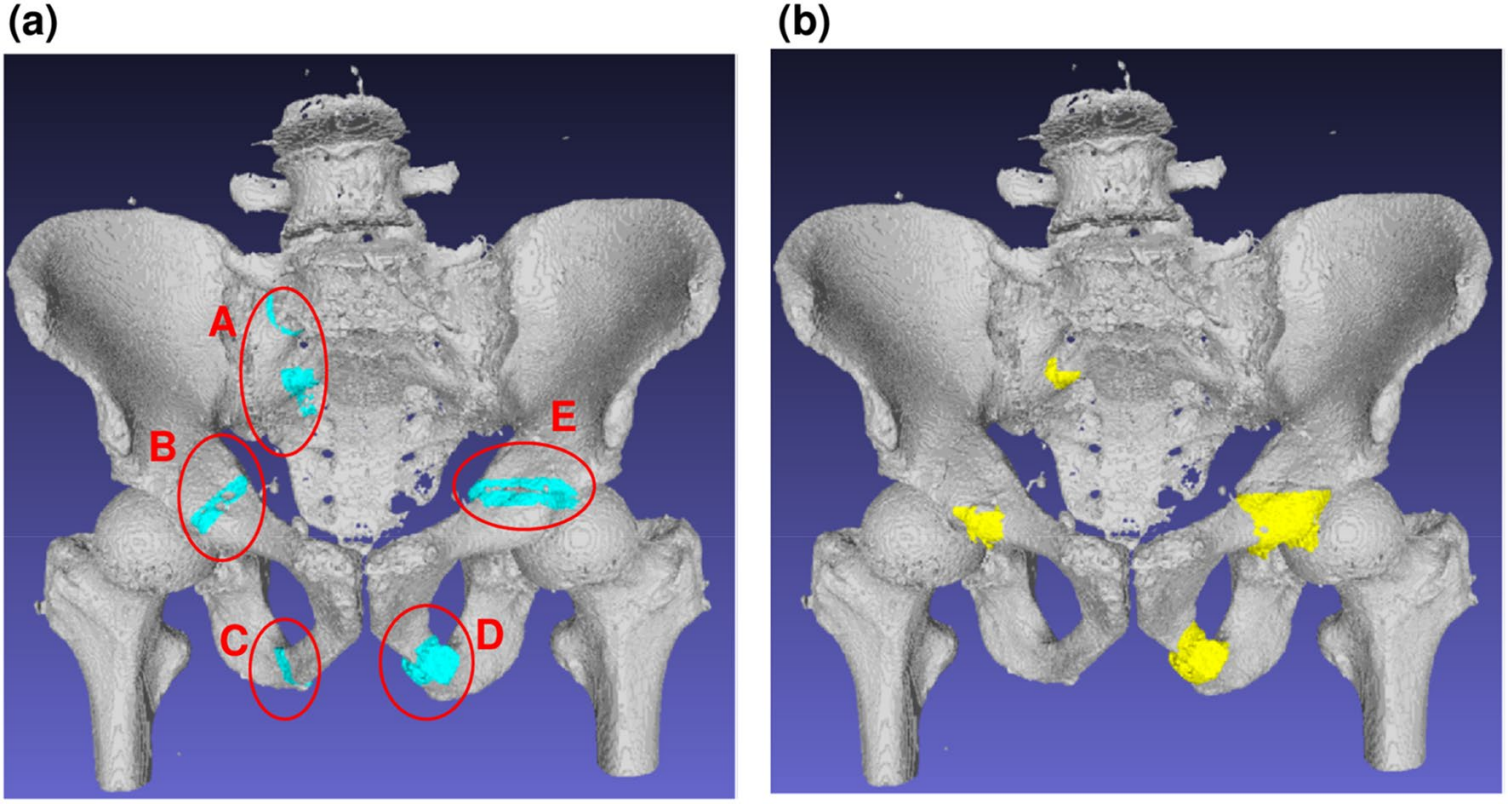
The 3D visualization of fractures. (**a**) Ground truth fractures. (**b**) Automatically detected fractures.

Figure 4 shows the comparison of ground truth fractures and automatically detected fractures under the proposed method. Fractures on the 3D bone surface are highlighted. The subject had five ground truth fractures (**A**–**E**). The proposed method successfully detected all except one fracture (**C**). The IoUs of **A**–**E** were 15.4, 7.3, 0.0, 30.3, and 15.0, respectively. Although the detected volume is slightly different from the ground truth fractures, the detected region is located close to the ground truth fractures, and will assist physicians in identifying the fractures.

### Subject-wise recall and specificity

Subject-wise recall and specificity were evaluated using dataset **A** with bone fractures, and dataset **B** without bone fractures, respectively. For comparison, we called the recall and specificity calculated for each fracture the fracture-wise recall and specificity. Only dataset **A** was used for training YOLOv3 models, and sixfold cross-validation was also conducted. Analysis parameters were: ${C}_{th}$ was 0.2, ${I}_{th}$ was 6, and the threshold of IoU was 10%. Subject-wise recall and specificity were evaluated for each subject (not for each fracture), where a positive subject denotes a subject in whom one or more fractures are detected, and a negative subject denotes a subject in whom no fractures are detected. Subject-wise recall calculated using dataset **A** was 1.00 (93 of 93 subjects), showing that the proposed method completely detected all subjects with bone fractures. The ratio of subjects for whom all fractures were detected was 0.559 (52 of 93 subjects). Subject-wise specificity for dataset **B** was 0.964 (4 of 112 subjects), and the proposed method successfully recognized all except 4 non-fracture subjects.

## Discussion

**Figure 5:**
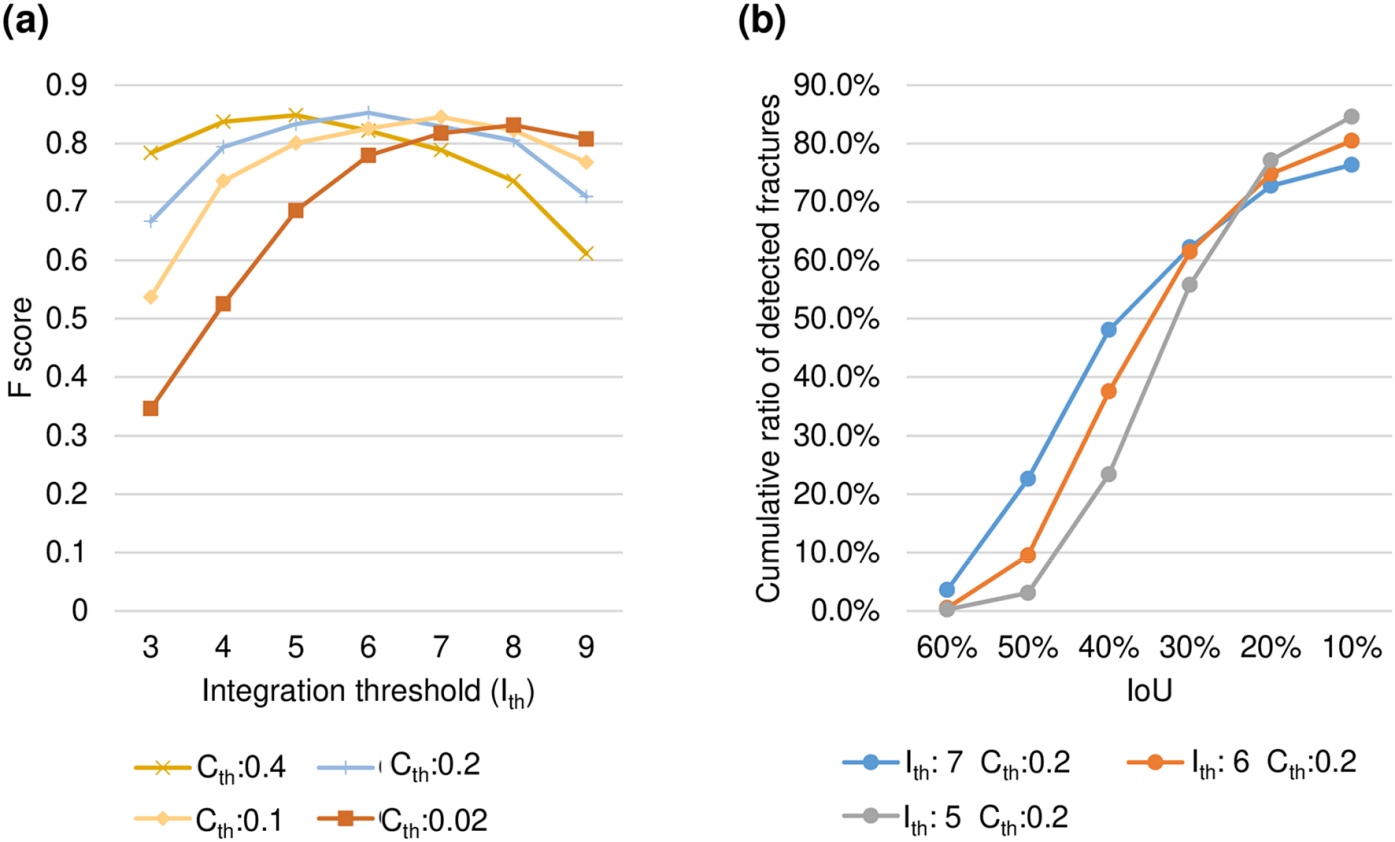
Performance dependency on analysis parameters. (**a**) Relationship between ${I}_{th}$ and ${C}_{th}$. (**b**) Cumulative histogram of IoU of the detected fractures.

The experimental results of the proposed method depend on parameters, ${C}_{th}$ and ${I}_{th}$. Figure 5a shows F score at IoU of 10% with changes in ${C}_{th}$ and ${I}_{th}$. For each of ${C}_{th}$= 0.4, 0.2, 0.1, and 0.02, the highest F score was 0.849 (recall 0.792, precision 0.914) at ${I}_{th}=5$, 0.853 (recall 0.805, precision 0.907) at ${I}_{th}$= 6, 0.845 (recall 0.810, precision 0.884) at ${I}_{th}$= 7, and 0.832 (recall 0.802, precision 0.863) at ${I}_{th}$= 8. A tendency was seen for ${I}_{th}$ to be decreased when ${C}_{th}$ was large, while the ${I}_{th}$ should be increased when ${C}_{th}$ is small. This is because that the number of fracture candidates detected by multiple YOLOv3 models in parallel increases with decreasing ${C}_{th}$, and can be suppressed by increasing ${I}_{th}$ at the integration step. Next, Fig. 5b shows a cumulative histogram of IoUs of the detected fractures by the proposed method with ${I}_{th}$= 5, 6, and 7. This shows that the ratio of high IoU fractures increased with higher ${I}_{th}$, because the integration of multiple orientation results specifies the fracture region more precisely.

**Figure 6:**
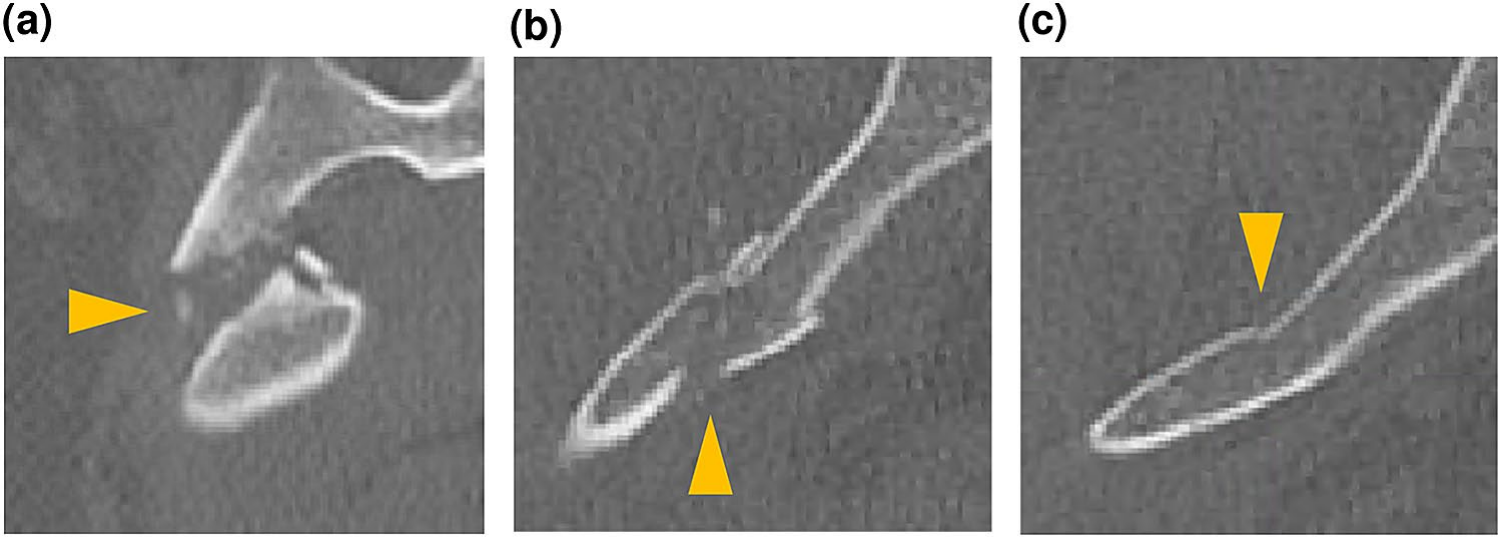
Types of fractures. (**a**) Completely displaced fracture (**F1**). (**b**) Incompletely displaced fracture (**F2**). (**c**) Compression fracture (**F3**). Fractures are indicated by triangles.

Next, detection accuracy among appearance types was discussed. We classified bone fractures into 3 types: (**F1**) completely displaced fracture; (**F2**) incompletely displaced fracture; and (**F3**) compression fracture. **F1** type represents fractures where fractured part of the bone is completely separated (Fig. 6a). **F2** type represents fractures where the fractured part of the bone is loosely separated (Fig. 6b). **F3** type represents the fractures where the fractured part of the bone is not separated but a part of the bone surface has changed (Fig. 6c). The 389 fractures of dataset **A** were classified into 67 **F1** fractures, 282 **F2** fractures, and 40 **F3** fractures. Figure 6 shows examples of axial CT images for the 3 fracture types. We calculated fracture-wise recall for each type of fracture using parameters with ${C}_{th}$ of 0.2 and ${I}_{th}$ of 6 that provided the highest F score. The fracture-wise recalls were 0.955 (**F1**), 0.869 (**F2**), and 0.350 (**F3**). The accuracy of **F3** type was lower than that of the other types, because few characteristics of fracture were present on the image.

Processing time for one subject was 756 s in total, using a computer with an i9-10900 k CPU, and a TITAN-RTX GPU. The method consists of 3 steps; the first step to synthesize slab image took 361 s, the second stage to predict 2D fracture candidates took 253 s, and the third stage to aggregate them into 3D space took 142 s.

The proposed method will assist physicians to detect pelvic fractures. While fracture detection performance will be increased, the risk of misleading physicians must be considered. Use of the method should thus be limited to second-stage interpretations after the first interpretation without the AI-system. A limitation to the proposed method is that it is not applicable to patients with implants. The future prospects for the proposed method includes extending the methods for patients with implants, compared with other object detection methods such as Faster R-CNN, SSD, and optimization of deep learning parameters.

## Subjects and materials

Two datasets were used in this study. Dataset **A** consists of CT images acquired from 93 subjects who had one or more pelvic fractures. Dataset **B** consists of CT images acquired from 112 subjects identified by orthopedic surgeons as not having any fractures. Both datasets were acquired at Steel Memorial Hirohata Hospital, Japan.

Dataset **A** was taken from 47 male and 46 female subjects with a mean age of 66.1 ± 18.9 years (range, 20–93 years). Each subject had one or more fractures of the pelvis, and no implant had been confirmed on CT images. Before subjects received surgical treatment, CT images were acquired using three multidetector-row CT (MDCT) scanners (SOMATOM Definition AS 32 line, SOMATOM Go. Top 64 line, or Sensation Cardiac 16 line; Siemens, Germany). The images were taken between April 2013 and August 2019. CT images covered the whole pelvis, and image acquisition parameters were: tube voltage, 120 kVp; current, auto mAs; spatial resolution, 0.61–0.98 mm; and thickness, 0.60–1.00 mm. No space between slices was used. All CT images were annotated by orthopedic surgeons for training and evaluation purposes. The annotation procedure is described in the following section. Dataset **A** was used for both training and evaluation.

Dataset **B** was taken from 69 male and 43 female subjects with a mean age of 61.3 ± 19.7 years (range, 20–93 years). No fractures or implants were confirmed on CT images by orthopedic surgeons specializing in pelvic fracture. CT images were acquired between July 2018 and December 2018 using an MDCT scanner (SOMATOM Definition AS 32 line; Siemens, Germany). CT images covered the whole pelvis, and image acquisition parameters were: tube voltage, 120 kVp; current, auto mAs; spatial resolution, 0.61–0.98 mm; and thickness, 0.70 mm. No space between slices was used. Dataset **B** was used for evaluation only.

The acquired CT images had 12-bit pixel resolution. As a preliminary step, CT values of 1–1,800 HU were linearly converted into 0–255. To normalize pelvic size, CT images were normalized into 296 × 169 × 288 mm, as the average size of the pelvis for 30 randomly selected subjects, using B-spline interpolation. The resulting dimensions were 494 × 282 × 480 voxels, and voxel size was 0.6 × 0.6 × 0.6 mm.

## Methods

The proposed method automatically detects the pelvic fractures from pelvic CT images of an evaluating subject with the following steps. First, nine different orientation images are synthesized from the raw CT images. For each orientation, the second step extracts 2D bone fracture candidates using trained DCNN models. The third step aggregates 2D bone fracture candidates in 3D space, then segments the 3D bone fracture region. The DCNN models are based on YOLOv3. An annotation method based on annotating 3D surface is also introduced. Detection performance is validated based on precision, recall, F-score and AUC. The details are described below.

### Multi-orientated image synthesis

**Figure 7:**
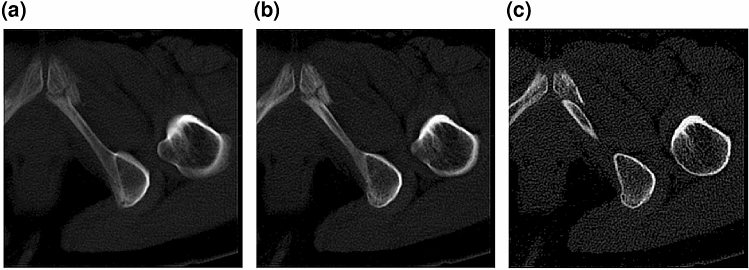
A 2.5D representation. (**a**) Image with 31-image thickness. (**b**) Image with 15-image thickness. (**c**) Image with 1-image thickness.

A volume of a subject is first divided into four cubes to reduce the memory required for YOLOv3 analysis. The dimensions of each cube are 282 × 282 × 282 voxels. Cubes are extracted from the edge of the normalized volume so that overlaps between cubes are minimal. The proposed method synthesizes 9 orientation images from each cube; the 9 orientations are 3 orthogonal directions and 6 diagonal directions. In the synthesized images, the out-of-imaging area is filled by 0. Next, for each sectional image, three slab images with 31-image (18.6 mm), 15-image (9.0 mm), and 1-image (0.6 mm) thickness are synthesized to represent neighboring information according to 2.5-D representation. Slab images are synthesized by averaging neighboring images. Figure 7 shows an example of three slab images.

### Bone fracture region extraction method

The proposed method simultaneously extracts 2D bone fracture candidates from multiple 2D orientation images. By aggregating 2D bone fracture candidates, the 3D bone fracture region is segmented. The procedure for bone region extraction is described below.

*Step 1* Detect bone fractures from 2D images using multiple YOLOv3 models in parallel. Nine YOLOv3 models are prepared to analyze 9 orientation images. For each orientation, a set of three thicknesses of slab images is fed to the input layer of YOLOv3 model, which then yields coordinates of bounding boxes and confidence scores. If the confidence score is greater than or equal to a threshold confidence score (${C}_{th}$), the bounding box is detected as a fracture candidate. The fracture candidates are thickened by 12.6 mm to cover the whole fracture volume, such as that for a completely displaced fracture (**F1**).

*Step 2* Integrate the thickened fracture candidates detected from the multiple YOLOv3 models into one 3D volume. Each voxel represents the degree of fracture, defined as the number of orientations included in the thickened fracture candidates. Each voxel takes a value between 0 and 9.

*Step 3* Segment the fracture region by thresholding the obtained 3D volume. The voxels with a value equal to or higher than ${I}_{th}$ are set to 1, and all others are set to 0. Small fracture regions in which the number of voxels is less than 8000 are discarded to suppress over-detection. The remaining regions are finally detected as fracture regions.

### Method of 3D surface annotation

**Figure 8:**
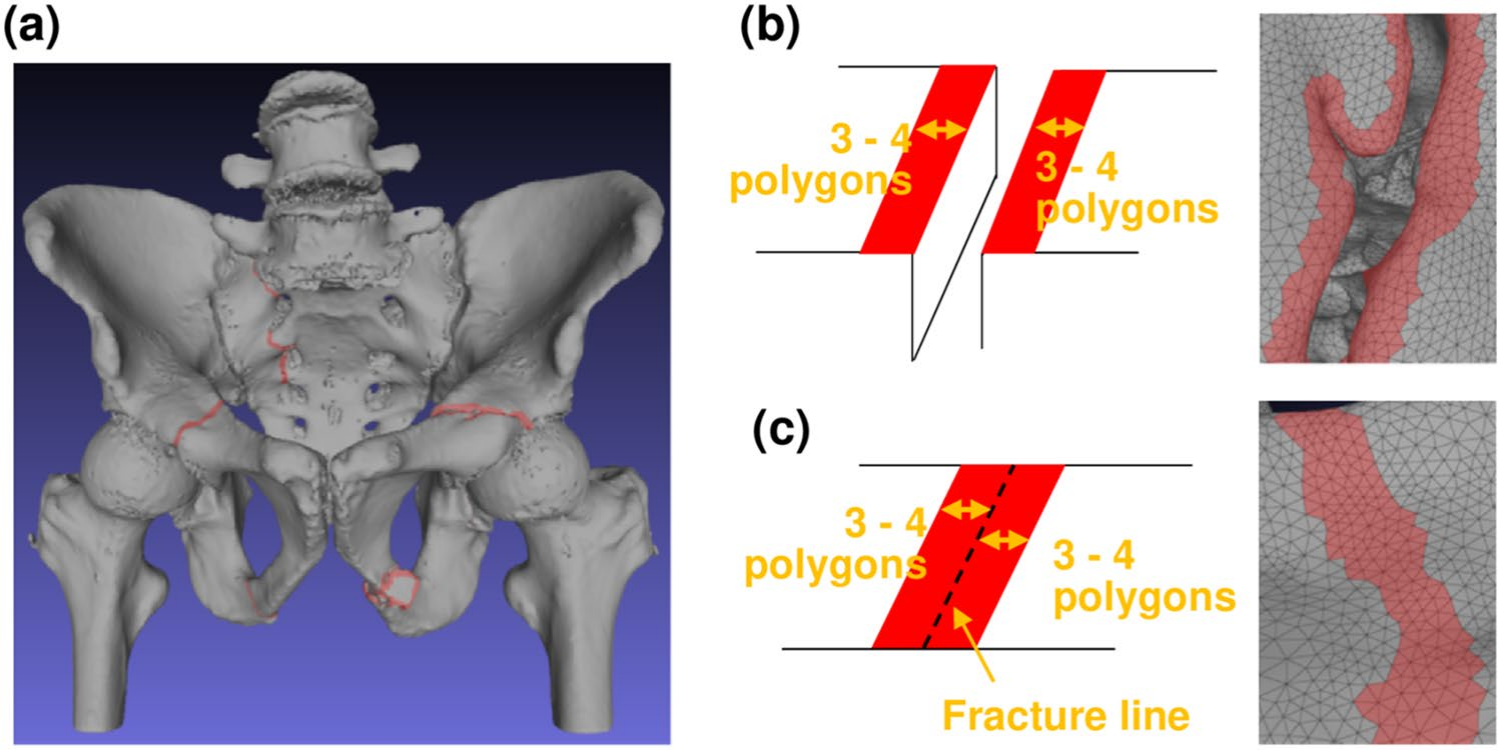
The 3D annotation method. (**a**) Annotated 3D bone surface data. (**b**) Annotation of completely displaced fracture (**F1**). (**c**) Annotation of incompletely displaced fracture (**F2**) or compression fracture (**F3**).

The proposed method requires annotation of bone fracture regions to train YOLOv3 models. However, the number of CT images is huge, and manually performing the annotation procedure that surrounds a fracture area with a polygon is too difficult. To annotate fractures efficiently, this study introduces a new 3D annotation scheme using 3D surface rendering. The 3D surface rendering is performed by representing the pelvic bone surface on CT images with a set of small polygons. The pelvic bone region is easily segmented using image processing such as thresholding, morphological operation, etc. Orthopedic surgeons select 3–4 adjacent polygons around fractures using the 3D surface rendering as shown in Fig. 8a. For example, a completely displaced fracture (**F1**) is annotated as shown in Fig. 8b. An incompletely displaced fracture (**F2**) or a compression fracture (**F3**) is annotated as show in Fig. 8c. After 3D annotation on the pelvic bone surface, the annotated 3D polygons are converted into 2D bounding boxes on sectional images for each orientation. Because YOLOv3 model evaluates the three slab images with 18.6 mm, 9.0 mm, and 0.6 mm thicknesses, the 2D bounding boxes are also thickened by 18.6 mm.

### YOLOv3 model training

A YOLOv3 model^27^ pre-trained with the ‘COCO trainval’ dataset is used. YOLOv3 model is trained using a set of three 2.5D images and the ground truth bounding boxes. A different YOLOv3 model is trained for each of the 9 orientation images, and 9 YOLOv3 models are obtained. The training data are augmented by brightness adjustment, rotation, horizontal flip, enlargement, reduction, and changing the aspect ratio. Each model is fine-tuned on three output layers for the first eight epochs with a learning rate of 0.001, then all layers are fine-tuned for the following ten epochs with a learning rate of 0.0001. The batch size is 28. The input size of the model is 416 × 416.

To correct the imbalance in the number of images with and without fractures, the volume of a subject is divided into volumes with 20 consecutives slices. For each divided volume, when the number of images with fractures over all subjects is less than 10%, 10% of images without fracture from the same volume are randomly chosen. Otherwise, the same number of images without fractures are selected randomly. For training the model, the multi-orientated synthesized images from dataset **A** are used. Table 1 shows the total number of synthesized images from dataset **A** for each orientation. The data were decomposed into 6 folds to perform 6-folds-cross-validation test.

**Table 1:** Total number of synthesized images from dataset **A** (93 subjects).

Orientations	With fracture	Without fracture
Orthogonal direction 1	31,217	62,527
Orthogonal direction 2	23,524	70,220
Orthogonal direction 3	29,766	63,978
Diagonal direction 1	29,298	107,970
Diagonal direction 2	33,942	103,326
Diagonal direction 3	28,011	109,257
Diagonal direction 4	30,385	106,883
Diagonal direction 5	32,697	104,571
Diagonal direction 6	29,083	108,185

### Evaluation metrics

Precision, recall, F-score, and AUC are calculated to evaluate the results. The ground truth of the 3D fracture region is prepared by intersections of 2D fracture boundary boxes at every orientation image. Then, IoU is calculated between the detected and ground truth 3D fracture regions. The IoU is defined by Eq. (1).

1 $$IoU=\frac{{A}_{m}\cap {B}_{n}}{{A}_{m}\cup {B}_{n}},$$

where ${A}_{m}$ is a set of ground truth fracture regions, and ${B}_{n}$ is a set of the detected fracture regions. The correspondence between ground truth and detected region is determined by maximizing IoU. When IoU is greater than or equal to a threshold, the ground truth region is successfully detected. Otherwise, the ground truth region is not detected.

Fracture-wise precision and recall are calculated using true positive (TP), false positive (FP), and false negative (FN). TP denotes the number of ground truth fractures successfully detected. FP denotes the number of fractures detected incorrectly. FN denotes the number of ground truth fractures that are not detected. Precision, recall, and F score are defined by Eqs. (2)–(4).

2 $$Precision=\frac{TP}{TP+FP}$$

3 $$Recall=\frac{TP}{TP+FN}$$

4 $$F score=\frac{2\cdot Recall\cdot Precision}{Recall+Precision}$$

The interpolated precision^33^ is calculated by sampling precision whenever it drops and computing the sum of the rectangular blocks using Eq. (5).

5 $${p}_{interp}\left({r}_{n}\right)=\underset{\tilde{r } : \tilde{r } \ge {r}_{n}}{\mathit{max}}\left(p\left(\tilde{r }\right)\right),$$

where $p\left(r\right)$ = precision at recall r.

### Ethical approval

Approval was obtained from the Institutional Review Board to conduct the study and the need to obtain informed consent from subjects were waived (Steel Memorial Hirohata Hospital IRB #1–52). These analyses were performed in accordance with the relevant rules, guidelines and regulations.

### Informed consent

The need for informed consent was waived by Institutional Review Board (Steel Memorial Hirohata Hospital IRB #1-52) due to the retrospective nature of the study.
